# Mesenchymal Stromal Cell-Derived Tailored Exosomes Treat Bacteria-Associated Diabetes Foot Ulcers: A Customized Approach From Bench to Bed

**DOI:** 10.3389/fmicb.2021.712588

**Published:** 2021-07-27

**Authors:** Alok Raghav, Prashant Tripathi, Brijesh Kumar Mishra, Goo-Bo Jeong, Shahid Banday, Kirti Amresh Gautam, Qazi Noorul Mateen, Prem Singh, Manish Singh, Akhil Singla, Jamal Ahmad

**Affiliations:** ^1^Multidisciplinary Research Unit, Department of Health Research, MoHFW, GSVM Medical College, Kanpur, India; ^2^Department of Biochemistry, GSVM Medical College, Kanpur, India; ^3^Department of Endocrinology, UCMS, GTB Hospital, New Delhi, India; ^4^Department of Anatomy and Cell Biology, College of Medicine, Gachon University, Incheon, South Korea; ^5^Department of Molecular, Cell and Cancer Biology, University of Massachusetts Medical School, Worcester, MA, United States; ^6^Department of Biochemical Engineering, Indian Institute of Technology Delhi, New Delhi, India; ^7^Department of Medicine, GSVM Medical College, Kanpur, India; ^8^Department of Neurosurgery, GSVM Medical College, Kanpur, India; ^9^Department of Medicine, Maharishi Markandeshwar College and Hospital, Maharishi Markandeshwar University, Solan, India; ^10^Faculty of Medicine, Rajiv Gandhi Centre for Diabetes and Endocrinology, JN Medical College, Aligarh Muslim University, Aligarh, India

**Keywords:** exosomes, diabetes foot ulcers, diabetes mellitus, customized exosomes, bacterial infection

## Abstract

Exosomes are nano-vesicles of endosomal origin inherited with characteristics of drug delivery and cargo loading. Exosomes offer a diverse range of opportunities that can be exploited in the treatment of various diseases post-functionalization. This membrane engineering is recently being used in the management of bacteria-associated diabetic foot ulcers (DFUs). Diabetes mellitus (DM) is among the most crippling disease of society with a large share of its imposing economic burden. DM in a chronic state is associated with the development of micro- and macrovascular complications. DFU is among the diabetic microvascular complications with the consequent occurrence of diabetic peripheral neuropathy. Mesenchymal stromal cell (MSC)-derived exosomes post-tailoring hold promise to accelerate the diabetic wound repair in DFU associated with bacterial inhabitant. These exosomes promote the antibacterial properties with regenerative activity by loading bioactive molecules like growth factors, nucleic acids, and proteins, and non-bioactive substances like antibiotics. Functionalization of MSC-derived exosomes is mediated by various physical, chemical, and biological processes that effectively load the desired cargo into the exosomes for targeted delivery at specific bacterial DFUs and wound. The present study focused on the application of the cargo-loaded exosomes in the treatment of DFU and also emphasizes the different approaches for loading the desired cargo/drug inside exosomes. However, more studies and clinical trials are needed in the domain to explore this membrane engineering.

## Introduction

**FIGURE 1:**
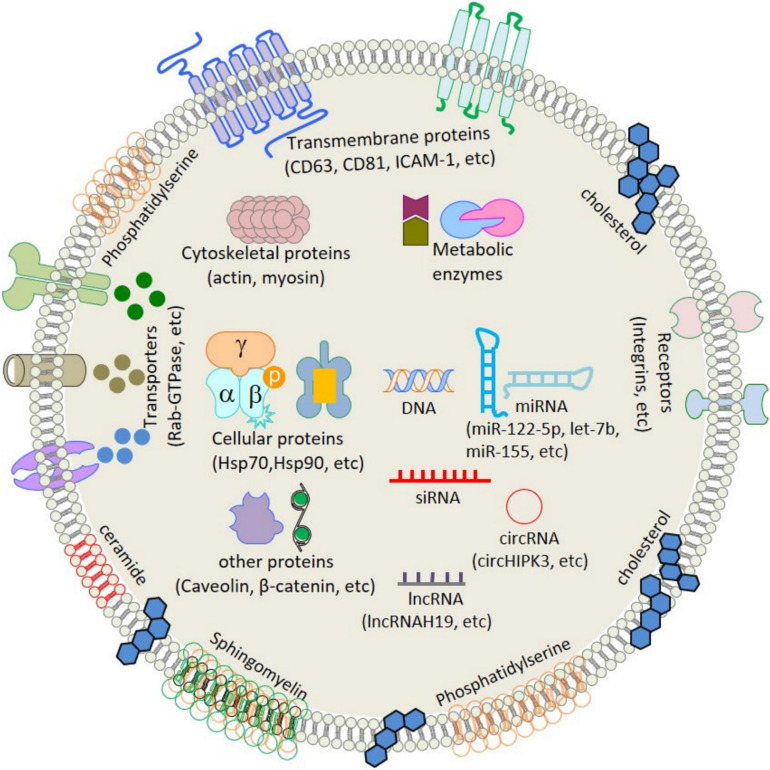
Schematic structure and contents of exosome. ATPase, adenosine triphosphatase; CD, cluster of differentiation; GAPDH, glyceraldehyde 3-phosphate dehydrogenase; HSP, heat shock protein; ICAM-1, intercellular adhesion molecule-1; LAM 1/2, lysosomal-associated membrane protein 1/2; MHC, major histocompatibility complex; miRNA, microRNA; mRNA, messenger RNA; MVB, multivesicular body; PGRL, PG regulatory-like protein; pgk1, phosphoglycerate kinase 1. [Adopted from Jan et al. (2021) distributed under the Creative Commons Attribution Licens].

Extracellular vesicles (EVs), including exosomes, apoptotic bodies, and microvesicles, are secreted by various cell types. EVs showed diverse characteristics in size, function, indigenous cargo, and secretion pathway (Raghav et al., 2021). Exosomes are small-sized EVs formed by the process of inward budding in early endosomes and later form multivesicular bodies (MVBs) of average 100-nm dimensions (Raghav et al., 2021). These later released into the extracellular matrix/environment to deliver their indigenous cargo/components fulfilling their fate (Raghav et al., 2021). Cellular exosomes release involves various steps, i.e., formation of early endosomes, followed by fusion of the MVBs containing intraluminal vesicles (ILVs), with the plasma membrane by exocytosis and release of exosomes in the extracellular space (Than et al., 2017). Exosomes are present in all bodily fluids secreted by cells, including blood (Lewis et al., 2018), urine (Cavallaro et al., 2019), plasma (Yan et al., 2019), breast milk (Adriano et al., 2021), saliva (Kurian et al., 2021), bile, synovial fluid, semen, amniotic fluid, ascites fluid (peritoneal cavity), and bronchoalveolar and gastrointestinal lavage fluid (Kumar et al., 2019). The exosomal indigenous cargo is mostly rich in proteins, lipids, sugars, and nucleic acids [messenger RNAs (mRNAs), microRNAs (miRNAs), and mitochondrial DNA (mtDNA), etc.] (Jan et al., 2021; Figure 1). Exosomes’ functions encompass an elaborative list depending on the origin of cell/tissue. Such functions include immune-modulatory, regeneration, antigen presentation programmed cell death (APPCD), inflammation, angiogenesis, and coagulation. The cargo imparts functionality to the exosomes for different cellular communications like paracrine, autocrine, endocrine, and/or juxtacrine signaling, while surface proteins provide identity to the exosomes for cargo delivery (Wei et al., 2021).

Authors of past studies exploited the exosomes as delivery vehicles for drugs and other desired cargo of interest (Bertrand and Leroux, 2012; Lai and Breakefield, 2012; El Andaloussi et al., 2013). These inbuilt characteristics of exosomes allow for tailoring “cargo of interest” for therapeutics and imaging purpose with an additional feature of prolonged circulation time, specific target cell recognition due to the presence of cell surface markers, negligible toxicity, and immune tolerance. Exosomes can be manipulated with more than one type of deliverables like drugs, proteins, and coding/non-coding nucleic acids, simultaneously. However, further studies are required to evaluate whether there exists any sort of allogeneic immune rejection among exosomes from different donors and recipients (Zhuang et al., 2011; Lee et al., 2012).

In one of the recently published studies, the protective effect of adipocyte stem cell (ADSC)-derived exosomes was investigated in a diabetic animal *in vitro* model and found that exosomes promoted angiogenesis and proliferation of cells in the hyperglycemic environment (Li et al., 2018). The study showed a significant reduction in diabetic ulceration/wound area in the animal group receiving the exosomes from ADSCs overexpressing the Nrf2 factor (Li et al., 2018). The study laid the foundation that the exosomes can be exploited for the healing of diabetic foot ulcers (DFUs). An et al. (2021) showed the therapeutic role of mesenchymal stem cell (MSC)-derived exosomes in the treatment of diabetes-induced ulcers and lower limb ischemia.

Diabetic foot ulcers are a severe complication associated with diabetes mellitus (DM) that impose economic burden ranges from US$9 to US$13 billion in the United States, along with additional cost for the management of DM (Raghav et al., 2018). DFUs are the cause of various complications including peripheral neuropathy, deformity in the foot, and peripheral arterial diseases’ poor extremity perfusion (Noor et al., 2018). DFUs are characterized by the presence of bacterial pathogens that are responsible for wound microbiology and the development of the infection. Several microorganisms (fungi, aerobic, and anaerobic species) are responsible for the etiology of the DFUs, including *Staphylococcus*, *Streptococcus*, *Proteobacteria*, and *Pseudomonas aeruginosa* (Noor et al., 2015). In this review, first, we comprehensively focused on exosome biogenesis and factors affecting the biogenesis. In addition, we discussed the methods of isolation of exosomes and fabrication of the customized exosomes using various modification methods. This study discusses the idea that MSC-derived exosomes post-tailoring hold promise to accelerate the diabetic wound repair in DFU associated with bacterial inhabitant, along with the application of the cargo-loaded exosomes in the treatment of DFU, and this study also emphasizes the different approaches for loading the desired cargo/drug inside exosomes.

## Biogenesis of Exosomes

**FIGURE 2:**
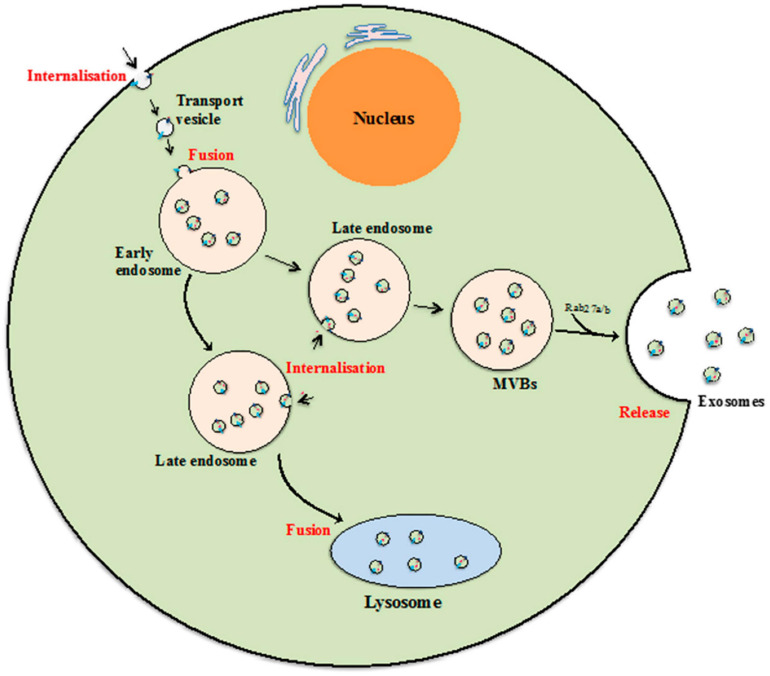
Exosome biogenesis. Beginning from internalization of membrane proteins and lipid complexes by endocytosis, endocytotic vesicles are delivered to early endosomes, which fuse with each other resulting in formation of late endosomes/multivesicular bodies (MVB). [Adopted from Than et al. (2017) distributed under the Creative Commons Attribution License].

Biogenesis of exosomes is a constitutive mechanism that is initiated with plasma membrane inward invagination within cytosol generating early and late endosomes. These late endosomes further give rise to MVBs followed by ILV formation. It seems that during the ILV formation by inward budding, several essential proteins, growth factors, cytoskeleton components, nucleic acids, lipids, and other necessary cellular components get wrapped into it (Raghav et al., 2021). The key feature of biogenesis pathways includes internalization, fusion, and release (Figure 2). ILVs formed from MVBs fuse with the plasma membrane of the cells and released as exosomes into the extracellular environment by the mechanism of exocytosis.

In one of the recently published studies, it was quoted that the budding of the exosomes and their sorting are either endosomal sorting complex required for transport (ESCRT)-dependent or -independent (Raghav et al., 2021). The ESCRT-mediated exosomes sorting process involves screening, identification, and sequestration of ubiquitinated proteins specific for endosomal proteins. This ESCRT-mediated mechanism showed an association between subunits I, II, and III of ESCRT that terminate the exosome budding process (Raghav et al., 2021). Moreover, the ESCRT-independent mechanism of exosome budding involves proteins and lipids such as tetraspanins and ceramides (Raghav et al., 2021). The exosomes play a crucial role in intercellular communication *via* the transfer of the biomolecules loaded within them. Their biogenesis mechanism is governed by various factors including ESCRT proteins, STAM1, VPS4, CHMP4, the Syndecan–syntenin–ALIX complex, nSMase2, CD9, and PLD2 (Gurunathan et al., 2021). Similar to the sorting mechanism, the exosome uptake process is mediated by either the clathrin-dependent or clathrin-independent events that involve micro-pinocytosis, phagocytosis, and lipid raft-mediated internalization. The exosomes are composed of several biomolecules including heat shock proteins, cell adhesion proteins, cell signaling proteins, tetraspanin membrane proteins, phosphatidylserine (PS), phosphatidic acid, sphingomyelin (SM), cholesterol, arachidonic acids, prostaglandins, and leukotrienes (Raghav et al., 2021). Besides these proteins and lipid components, exosomes are also rich in micro-RNAs, small nuclear RNAs, non-coding RNAs, long non-coding RNAs, piwi-interacting RNAs, rRNAs, and tRNAs (Raghav et al., 2021). Exosomes are considered to be the cocktail of these biomolecules that have therapeutic, diagnostic, and transmittance characteristics.

## Sources of Exosomes

Exosomes can be derived from various cell types and all have diverse clinical characteristics, depending on the source of cells from which they are derived. The various sources of exosomes include the following:

### ADMSC-Derived Exosomes

Adipose tissue mesenchymal stem cells (ADMSCs) are abundantly distributed in the human body, compared to other exosome cell sources including umbilical cord mesenchymal stem cells (UCMSCs) and bone marrow mesenchymal stem cells (BMSCs). ADMSCs showed the highest degree of purification with high yield due to their abundance in nature (Tang et al., 2021). The extraction of ADMSCs is an easier and painless procedure, causing only a small episode of trauma (Tang et al., 2021). These cell-derived exosomes can be easily procured in clinics in the presence of plastic surgeons or medical aestheticians. ADMSC-derived exosomes require strict storage conditions, thereby possessing some restrictions in their clinical applications. Moreover, the lipid membrane structure is quite stable and has the properties to retain its contents for a long period and is therefore always a good choice for researchers (Tang et al., 2021).

The strict morphology of the adipose tissue is not fixed, and the primary source cells can be of any shape, either fusiform or circular. It was reported from *in vitro* cell culture observation that the primary cells get to adhere with other cells within 1–2 days of the cell culture (Tang et al., 2021). It was shown that after the fifth passage, these cells form a single layer, thereby showing a vortex or radial growth pattern (Tang et al., 2021). Later, their morphology changes to a single long spindle. The exosomes derived from ADMSCs have uniform cup shape morphology with average diameter ranges between 30 and 120 nm as evident from the scanning electron microscopy (Tang et al., 2021). The exosomes can be detected using flow cytometry, differential centrifugation, magnetic bead assay, and transmission electron microscopy. The presence of surface proteins CD9, CD10, CD13, CD29, CD44, CD63, CD73, CD90, CD105, enkephalin enzyme, and major histocompatibility complex MHC I molecules distinguishes these from other cell-derived exosomes. It is still a research lacuna that no single surface marker has been identified for these exosomes.

### UCMSC-Derived Exosomes

The umbilical cord has a placental origin and is involved in giving nourishment and nutrition to the fetus from the mother during pregnancy. Human-derived MSCs can be broadly classified into (i) human umbilical cord mesenchymal stem cells (hUC-MSCs), (ii) human umbilical cord perivascular MSCs (HUCPV-MSCs), (iii) human umbilical cord Wharton’s Jelly MSCs (HWJ-MSCs), and (iv) human amniotic membrane-derived MSCs (HA-MSCs) (Subra et al., 2010).

The morphology of the primary UCMSCs demonstrates a spindle shape with the absence of vortex growth. Later, the cells show vortex growth after day 1 of the direct adherent cell culture using the primary cell tissue mass culture method. Moreover, few cells during the fifth passage show vortex patterns from the second generation until the fifth generation (Tang et al., 2021). With the fifth passage, the cells become long, elongated, and fusiform with typical vortex growth. The exosomes derived from these primary cells show variation in size in the range between 30 and 100 nm, as revealed from electron microscopy (Tang et al., 2021). The exosome morphology exhibits a round or elliptical membranous structure with clear and distinct boundaries (Vohra et al., 2020). The UCMSCs exhibit cell-specific surface markers including CD29, CD44, C/73 (SH3), CD90 (Thy-1), and CD105 (SH2) and negative for CD11b, CD34, and CD45, while their extracted exosomes demonstrate CD9, CD63, and CD81 and the multivesicular biosynthesis-related protein ALIX (Tang et al., 2021).

### BMSC-Derived Exosomes

The BMSCs can be isolated from bone marrow with the inbuilt advantage of low infection rate of pathogenic microorganisms, efficient and stable biological role, low immune rejection post-transplantation, and good survival rate in higher passages (Tan et al., 2020). These cells exhibit diverse size and shape and become adherent after 1–2 days of the cell culture seeding in the appropriate culture medium. The adherent cell shows round morphology as demonstrated by electron microscopy. These cells begin to colonize after 4–5 days of the culture exhibiting a single fusiform shape forming a vortex growth pattern usually at the fourth passage (Tang et al., 2021). The exosomes derived from BMSCs are uniform with a size range between 30 and 100 nm in diameter and having a cup-shaped morphology with clear and distinct boundaries (Tang et al., 2021).

Western blotting and flow cytometry analysis of the BMSC-derived exosomes show expression of CD9, CD63, CD81, HSP70, syntenin-1, and multi-vesicular biosynthesis-related protein TSG101 (Tang et al., 2021).

## MSC-Derived Exosome Isolation Methods

The following exosome isolation methods are currently available worldwide: microfluidics, differential centrifugation, precipitation, antibody affinity capture, ultrafiltration, flushing separation, magnetic bead-based capture, and size-exclusion chromatography (SEC).

### Microfluidics

Microfluidics provides highly efficient, precise control, and rapid methods for isolating the exosomes on a single chip with manipulated fluids at microscale levels. The basic principle of microfluidics is that it manipulates a small quantity of the fluid using specialized micro-dimension channels using capillary forces. Its manipulation characteristic with fluids in a micro/nanoscale environment makes it a highly preferred method of choice among researchers. The basic design of this method involves a single chip of a few square centimeters dimension with a scope of scaling up isolation and separation. This unique approach relies on interdisciplinary sciences that include physics, fluid chemistry, micro-processing, and bioengineering. In one published study, a microfluidics chip is coupled with acoustic, electrophoretic, and electromagnetic separations, which showed a fast and efficient way of exosome isolation and separation (Popovic et al., 2018). In another related study, the implication of silicon nanowires is engraved on the microchip pillar walls for trapping liposomes, and acoustic nanofiltration is used for isolation of exosomes within a size range of 100–1000 nm (Kurian et al., 2021).

One more study exhibited the use of viscoelastic microfluidics for the isolation and separation of the exosomes with an isolation efficiency of >80% and a purity degree of >90% (Kurian et al., 2021). Membrane of different pore sizes was also implicated for the separation of exosomes based on filtration using ExoTIC microfluidics chip (Lin et al., 2020). In another study, electric forces are applied along with a dialysis membrane of 30 nm pore size for the isolation of exosomes (Yang et al., 2017). Wu et al. (2017) used whole blood to isolate exosomes using the acoustic fluidics approach in combination with microfluidics. This system showed the unique feature of the cell removal module, which separates exosomes from microvesicles (Wu et al., 2017). The main advantages of this method are as follows: (i) it requires a lower amount of the sample volume, (ii) it is a time-saving approach, and (iii) it is a cost-saving and real-time process. The only disadvantage of this method is less sensitivity for the isolation of exosomes. So, a scale-up is required in this technology for the production of clinical-grade exosomes.

### Differential Centrifugation

This is the most widely used method for the isolation of the exosomes (Momen-Heravi et al., 2013). Cell debris and apoptotic bodies shed exosomes during successive rounds of the centrifugation mechanism. This method is based on the density, size, and shape of the exosomes. This gold standard method for exosome isolation, however, exhibits low yield and insufficient purity due to similarity in sedimentation properties of the different types of EVs (Tauro et al., 2012; Witwer et al., 2013; Cvjetkovic et al., 2014; Lane et al., 2015).

The main advantages of this method include reduction of cost and contamination. Additionally, a large sample capacity can be easily handled with this technique followed by high yields of exosomes. In another study, researchers have added 30% sucrose in the first step and reported a high yield of the exosomes (Bajimaya et al., 2017). Moreover, the limitations of the present approach are that high-speed centrifugation can damage the exosomes and it needs a long runtime with labor-intensive work. In one of the studies, it was found that performing ultracentrifugation three times reduces the purity of the exosomes (Tang et al., 2021).

### Precipitation

The precipitation of the exosomes depends on the principle of altering the solubility or dispersibility of the exosomes within a water-devoid medium. In this approach, the external solvent is implicated in the solution, which changes the polarity and solubility of the components present within the components, as a resultant, initiate the precipitation of desired molecules. It is a very simple approach for the isolation of the exosome. In one of the previously published studies, it was found that the precipitation approach is very effective in the separation of biological fluids (Maroto et al., 2017). Several commercial isolations and purification kits for exosomes are available, showing good yield and purity, including Serum^TM^, the Exo-Q and Exo-Spin^TM^ blood cell purification kits, the mi-RCURY Exosome Separation Kit, the Exo Quick-TC Exosome^TM^ Precipitation Solution Kit, and the Total Exosome Isolation kit (Maroto et al., 2017; Zhao et al., 2017; Buschmann et al., 2018; Soares Martins et al., 2018). The advantages of the current methods are that they are easy to use, do not require sophisticated and specialized machines, do not put any harsh effect on exosomes, and can be used on large sample volumes. Some limitations of these methods include the co-precipitation of other contaminants like polymeric materials, proteins, and lipids and the fact that they additionally require a long runtime to complete the process.

### Magnetic Bead-Based Capture

This process is also called an immunomagnetic bead-based assay. It is a recently developed technology that uses ExoCAS-2 charge-based ion exchange and magnetic beads for the isolation of exosomes from biofluids (Kim and Shin, 2021). This ExoCAS-2 implicates polycationic polymer-functionalized and -coated magnetic beads. The sample before the magnetic separation is filtered to exclude the large size impurities present. The mechanism of separation of the exosomes involves the binding of negatively charged exosomes with the positively charged poly-L-lysine-coated cationic beads *via* electrostatic interactions (Kim and Shin, 2021). Following the process of incubation and continuous stirring, the nano-sized exosomes bind to the surface of the coated beads and later eluted using an elution buffer with different ionic strengths that disrupts the electrostatic interactions. This efficient exosome separation and isolation approach yields exosomes of high purity grade, but the limitations associated with this technology are that it cannot be used in clinics, it has a high cost, and the rate of unspecific binding during the binding process is higher.

### Ultrafiltration

This technique is based on the application of specific pore size diameter membranes for separation and isolation of the exosomes (Cheruvanky et al., 2007; Lobb et al., 2015; Konoshenko et al., 2018). This approach can be complementary with ultracentrifugation, although it can also be performed alone. Another improved version of ultrafiltration includes cross-flow filtration or tangential flow filtration (McNamara et al., 2018). This improvement helps in removing the protein contaminants from the exosomes containing samples if repeatedly passed from the exclusion filter of a defined diameter, thereby concentrating the exosomes. In one of the studies, it was claimed that a cellulose membrane with a pore size of 10 kDa is very efficient in the recovery of the exosomes using an ultrafiltration approach (Vergauwen et al., 2017). The advantages of ultrafiltration are that it does not require expensive equipment and consumes less time. The only associated limitation with the ultrafiltration method is exosome loss due to attaching with membranes as a result of shear stress and membrane clogging.

### Size-Exclusion Chromatography

Size-exclusion chromatography depends on the separation of the exosomes’ molecules based on their size. The sample containing the exosomes is passed through the column consisting of the beads with variant pore size. Each molecule is passed through the individual beads based on their size. The small-size molecules show delayed elution from the column, as they have to traverse the complete length of the column. In one of the studies, it was found that exosomes have large hydrodynamic radii, passing through the column faster, as they do not show penetration inside the beads (Feng et al., 2014). In another, a single-step SEC using a Sepharose CL-2B column was used for isolation of exosomes with 75 nm diameter effectively from body fluids (Böing et al., 2014). This method allows minimal harm to the isolated exosomes compared to other precipitation-based methods. The SEC approach for isolating exosomes can efficiently remove the plasma proteins from the biological samples, as claimed by one of the studies (Gámez-Valero et al., 2016). In one of the studies, the authors have isolated clean and non-aggregated exosomes with a size range of 50–200 nm (Hong et al., 2016). It is also evident that SEC in conjugation with an ultracentrifugation approach can be efficiently used for the isolation of the exosomes from the biological fluids, compared to alone itself. The main advantages associated with SEC are that it can be used for the separation of the small and large molecules in biological fluids without altering the exosomal structure. The only limitation is the requirement of a long runtime.

## Tailoring Approaches for MSC-Derived Exosome Modifications

Exosome-based delivery approaches showed promising benefits related to specificity, safety, and stability due to their inbuilt homing characteristics that exhibit effective delivery of desired cargo to specific target sites. Recent studies showed that exosomes can be used to deliver small interfering RNA (siRNA) or active pharmaceutical agents like drugs and vaccines to treat diseases (Aryani and Denecke, 2016). These nano-size envelopes tend to avoid phagocytosis and engulfment by lysosomes with a low immune response (Ha et al., 2016). Several tailoring approaches for modification of exosomes and loading of the desired cargo into the exosomes were studied, which can be broadly classified into two strategies: (i) exogenous tailoring of exosomes post isolation and (ii) endogenous tailoring during biogenesis of exosomes. Exogenous tailoring approaches can be further divided into an active and passive form; the active approach involves the sonication, extrusion, freeze–thaw cycles, electroporation, and chemical-based approach, while the passive form involves the incubation process. Moreover, the endogenous tailoring of exosomes involves the introduction of the cargo of interest into the cells producing exosomes, which commonly implies the application of transfecting cells with expression vectors as in genetic engineering for targeted therapy (Van der Meel et al., 2014). The following paragraphs provide a brief overview of the tailoring approach for modifications of exosomes.

### Exogenous Tailoring of Exosomes

#### Incubation

This tailoring approach simply involves the incubation of exosomes with the desired interest of cargo, which can be referred to as passive loading. The potential difference created due to the interplay between the concentration of desired cargo inside and outside the exosomes drives the infusion of desired cargos through the lipid bilayer membrane of exosomes. In few cancer-related research studies, this method was used to load chemotherapeutic drugs like paclitaxel and doxorubicin into the exosomes and also to observe enhanced chemotherapeutic effects (Tian et al., 2014; Yang et al., 2015; Salarpour et al., 2019). This enhanced effect of drug-loaded exosomes is observed due to the ease of crossing the blood–brain barrier. In another study, exosomes loaded with enzymes were used in the treatment of Parkinson’s disease (Haney et al., 2015). In one of the studies, the authors have co-incubated curcumin with exosomes and found that it gets self-assembled with the lipid bilayer of exosomes due to the interplay of hydroscopic interactions. The curcumin encapsulated exosome not only increased the target specificity but also enhanced the anti-inflammatory property of curcumin (Sun et al., 2010). Though proven to be useful for modifying exosomes for their enhanced functionality with the desired cargo, this method sometimes affects the size of exosomes resulting in low yield, low entrapment, and uncontrollable drug loading. The present method is simple, cost-effective, and effective in transporting hydrophilic cargos efficiently into the exosomes.

#### Sonication

Sonication provides an additional advantage of enhancing the loading of desired cargo inside the bilayer membrane of the exosomes. This approach utilizes sound waves generated from a sonicator machine to induce a shearing force effect upon the exosome membrane, which, in turn, increases the uptake of desired cargo inside the exosomes (Kim et al., 2016). Kim et al. (2016) successfully loaded paclitaxel and doxorubicin into the exosomes implicating this approach. It is believed that the sonication process decreases the micro-viscosity of the exosomal membrane that allows the passage of cargo inside (Kim et al., 2016). This cargo loading approach is healthy for biological molecules like small RNAs due to its high loading efficiency. Some limitations like the development of shearing forces, exosomal membrane deformation, heat generation during the sonication cycle, loss of exosomal surface proteins, and non-suitability for hydrophobic drug delivery are associated with this approach.

#### Extrusion

This tailoring method involves lipid bilayer membrane disruption of exosomes during extrusion through a small-size polycarbonate porous membrane. This reversible disruption in the membrane allows the entry of desired cargo of interest inside the exosomes (Haney et al., 2015). Le Saux et al. (2020) have reported that extrusion is an efficient and promising method for tailoring the exosomes and loading the desired cargo inside it for targeted delivery. In one previous study, exosomes were extruded with porphyrins (Fuhrmann et al., 2015). The extrusion mechanism reshapes and reforms the exosomal membrane extensively and thereby showed higher loading efficiency (Jamur and Oliver, 2010).

#### Freeze–Thawing

This tailoring approach involves freezing and subsequent thawing of the exosome sample in a vessel of desired cargo to be loaded. The mix is incubated at 37°C followed by rapid freezing at –80°C; the same steps were repeated several times depending on the efficiency of the system. It is always advisable to strictly monitor the freeze–thawing steps, because it may form the aggregates of these vesicles into large size particles (Haney et al., 2015; Le Saux et al., 2020). In one previously published study, catalase was loaded to exosomes using the freeze–thaw method (frozen at –80°C and thawed at RT) (Haney et al., 2015). It was also demonstrated that several freeze–thaw cycles resulted in a lipid dilution ratio that may be easily interpreted from fluorescence resonance energy transfer (FRET) assay (Sato et al., 2016). Though this method produces aggregates of exosomes with lower drug loading capacity compared to sonication or extrusion procedures, it is followed widely for cargo loading.

#### Electroporation

Tailoring of exosomes for loading cargo using electroporation is a commonly applied method that employs an electric field for cargo uptake. In the electric field, the phospholipid bilayer membrane is disrupted, thereby allowing the entry of hydrophilic compounds like small DNAs, miRNA, and siRNAs (Faruqu et al., 2018; Kobayashi et al., 2020; Lv et al., 2020; Orefice, 2020). A recent study has reported that exosomes tend to form aggregates during electroporation, although it did not affect the function of exosomes. However, certain refinements in the technique such as carrying out electroporation in an optimal buffer containing trehalose maintain the structural integrity of exosomes (Johnsen et al., 2016). In another study, fused exosomes were derived from αv-integrin-specific iRGD peptide with doxorubicin efficiently with electroporation and proved targeted tumor therapy (Gong et al., 2019). In one of the previously published studies, it was found that miRNA delivery to exosomes under mild electroporation protects miRNA from RNase degradation and showed efficient loading (Pomatto et al., 2019). In light of all the studies, it can be said that electroporation is a reliable method for cargo loading in exosomes that preserves the naïve cargo without compromising the structural integrity of exosomes.

#### Chemical Transfection

Chemical transfection is preferably used to incorporate siRNA into exosomes under the influence of the transfection agent Lipofectamine 2000. Wahlgren et al. used a liposome-based transfection reagent to incorporate MAPK-1-siRNA into the exosomes by incubating at 37°C for 10 min (Wahlgren et al., 2012). This method of loading desired cargo into exosomes achieves relatively high transfection efficiency using lipids. Cationic transfection agents are the preferred choice of researchers considering their high degree of success. These chemical transfection reagents showed a high success rate in *in vitro* experiments; however, they have worse efficiency than electroporation. Immunogenicity and toxicity are some of the associated limitations of this approach.

### Endogenous Engineering-Based Tailoring of Exosomes Producing Cells

Genetic engineering is another remarkable approach for the production of loaded exosomes with desired characteristics and functions. This approach involves transfection of the donor cells, thereby initiating the upregulation of specific genes, allowing the synthesis of specific gene-linked cargo-loaded exosomes during their biogenesis. The insertion of the desired “gene of interest” in the parent cell type is achieved by either viral/non-viral invasion/infection. The infection efficiency is optimized by the quantity and quality of the exosomal cargo. It is well reported that exosomes originate through the endosomal machinery of the cell membrane. Exosomal content reflects lineage and the original cell type; therefore, depending on the experimental requirement and/or therapeutic applications, the host cell selection should be performed. Genetic engineering for modification of the exosomal content from different cell types predominantly involves two types of viral vectors: (i) retroviral and (ii) adenoviral.

Jiang et al. (2020) observed the therapeutic effect of tumor necrosis factor (TNF)-stimulated gene-6 (TSG-6) modified MSC-derived exosomes in a wound model and found that tailoring of such exosomes prevents scar formation. In addition, several research studies demonstrated the therapeutic role of MSC-derived exosomes tailored with such methods carrying miRNA in improving treatment modalities (Xin et al., 2012). Wei et al. (2019) successfully engineered immature mouse dendritic cells, for exosome production, expressing Lamp2b fused to αγ integrin-specific iRGD peptide for breast cancer treatment *in vitro*. In one of the studies, engineered HEK293T was used for expression of Lamp2B along with a fragment of IL-3 and showed a reduction in tumor growth and was found to be effective in treating chronic myeloid leukemia (CML) (Bellavia et al., 2017). Rivoltini et al. (2016) transduced K562 cells with lentiviral human membrane TRAIL (TNF-Related Apoptosis-Inducing Ligand) for the production of TRAIL (+) exosomes. The authors reported apoptosis in cancer cells on treatment with TRAIL exosomes. Furthermore, the *in vivo* analysis revealed that engineered exosomes induced necrosis and vessel damage in melanoma tumor subjects (Rivoltini et al., 2016). In another study, exosomes enriched with miR-503 showed promising therapeutic potential for cancer treatment (Bovy et al., 2015).

“Omni Spirant” (patent pending) is a recently developed regenerative gene therapy for cystic fibrosis (CF) and involves the use of surface-engineered exosomes/bioengineered stem cell exosomes. The method involves mucus penetration of the exosomes and delivery of the gene therapy cargo for the effective treatment of CF (Health Europa, 2021). Bioengineering of cells for the production of engineered exosomes has gained significant attention in the past few years. However, further studies are mandatory for designing protocols with improved stability, drug solubility, and bioavailability, for the therapeutic application of engineered exosomes.

## Therapeutic Role of Tailored MSC-Derived Exosomes in Bacteria-Associated DFU

Mesenchymal stromal cell have a diverse role including multi-differentiation and immunomodulation that significantly contribute in reducing inflammation-related complications (Philipp et al., 2018). These MSCs show a contributory role in a paracrine manner mediating through secreted growth factors, cytokines, and exosomes (Phinney and Pittenger, 2017). One of the previously published studies quoted that MSC-mediated paracrine secretion promotes wound healing (Kourembanas, 2015). The advantage of using exosomes over cell-based therapies is that these vesicles may overcome the side effects associated with cell transplantation such as immune rejection. Pathogenesis of bacteria-associated DFUs is contributed by poor innervation and vascularization and chronic inflammation. In a recent study, it was observed that exosomes derived from MSCs inhibit M1 polarization and simultaneously promote M2 polarization that helps in the reduction of the inflammation (Cao et al., 2017). It is also found that these exosomes promote skin wound healing mediated by the regulation of M2 polarization (Cao et al., 2017). This dual nature of exosomes, i.e., anti-inflammatory and skin wound healing, can be explored in bacteria-associated DFUs.

Tailored MSC-derived exosomes possess promising result in the treatment of DFUs and diabetic wounds. In a recent study, exosomes derived after pre-treatment of MSCs with salidroside (glucoside of tyrosol) showed healing of diabetic wounds (Ariyanti et al., 2019). Similarly, fluoxetine and pretreated MSC exosomes managed diabetic neuropathy well (Abdelrahman et al., 2018).

It has been proved that these exosomes occupy the class of paracrine factor that mediates the therapeutic, tissue repair, and wound healing effects of MSCs (Joo et al., 2020). Several clinical trials showed the efficacy of BMSCs in the treatment of diabetic wound and ulcers (Table 1). In another research, tailored exosomes derived from pretreated BMSCs with atorvastatin (ATV) showed an acceleration in the healing of diabetic wound both *in vivo* and *in vitro* (Yu et al., 2020). It has been found that pretreated BMSCs with ATV secrete exosomes that activate the AKT/eNOS signaling mechanism that further initiates the angiogenesis of endothelial cells mediated through upregulation of miR-211-3p, thereby showing significant wound healing in the diabetic environment (Joo et al., 2020). In another study of exosome modification, it was found that exosomes derived from blue light-exposed human umbilical cord MSCs showed improved wound healing mediated through upregulation of MEF2C signaling (Yang et al., 2019).

**TABLE 1:** Clinical trials of BM-MSCs in DFUs.

Cellular type	Object	Delivery method	Duration of observation	Clinical parameters
Autologous BM-MSCs	24 patients with non-healing ulcers of the lower limb (diabetic foot ulcers and Buerger disease)	Autologous cultured BM-derived MSCs along with standard wound dressing	12 weeks	Decrease in wound size, increase in pain-free walking distance, maintain normal liver and renal function, improve leg perfusion sufficiently
Autologous BM-MSCs	51 patients with impending major amputation due to severe critical limb ischemia	Intramuscular transplantation	6 months	Improve leg perfusion sufficiently to reduce major amputations and permit durable limb salvage, reduce analgesics consumption, increase in pain-free walking distance
Autologous biograft composed of autologous skin fibroblasts on biodegradable collagen membrane (Coladerm) in combination with autologous BM-MSCs	Patients with diabetic foot	Directly to the wound and injected into the edges of the wound, finally covered with prepared autologous biograft, received two additional treatments with cultured MSC on days 7 and 17	29 days	Decrease in wound size and an increase in the vascularity of the dermis and in the dermal thickness of the wound bed
Autologous BM-MSCs	41 type 2 diabetic patients with bilateral critical limb ischemia and foot ulcer	Intramuscular injection	24 weeks	Increase in pain-free walking distance, improve leg perfusion, ankle-brachial index (ABI), transcutaneous oxygen pressure (TcO_2_), magnetic resonance angiography (MRA) analysis
Autologous BM-MSCs	96 patients with critical limb ischemia and foot ulcer	Inject into the ischemic limb along the posterior and anterior tibial artery	120 days	79% limb salvage in patients

*Adopted from Cao et al. (2017) distributed under the Creative Commons Attribution License.*

**FIGURE 3:**
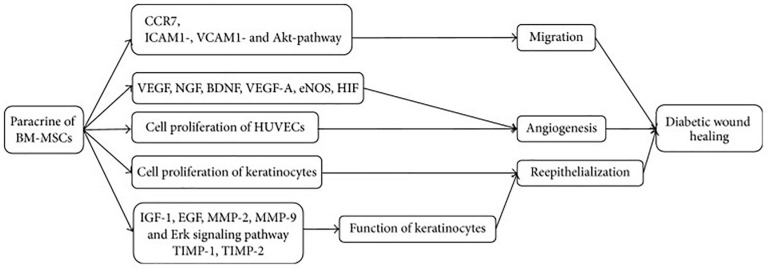
Mechanism of BM-MSCs for treatment of DFU. BM-MSCs can migrate and adhere via CCR7, ICAM1-, VCAM1-, and Akt- dependent mechanism and enhance angiogenesis through increasing VEGF, NGF, BDNF, VEGF-A, eNOS, and HIF. Cell proliferation of HUVECs and keratinocytes plays significant role in angiogenesis and reepithelialization, respectively. Keratinocyte function is improved by regulating IGF-1, EGF, MMP-2, MMP-9, TIMP-1, TIMP-2, and Erk signaling pathway. CCR7, C-C chemokine receptor type 7; ICAM1, intercellular adhesion molecule 1; VCAM1, vascular adhesion molecule 1; VEGF, vascular endothelial growth factor; NGF, nerve growth factor; BDNF, brain-derived neurotrophic factor; VEGF-A, vascular endothelial growth factor A; eNOS, endothelial nitric oxide synthase; HIF, hypoxia inducible factor; IGF-1, insulin-like growth factor 1; EGF, epidermal growth factor; MMP-2, matrix metalloproteinase-2; MMP-9, matrix metalloproteinase-9; TIMP-1, tissue inhibitor of metalloproteinase-1; and TIMP-2, tissue inhibitor of metalloproteinase-2. [Adopted from Cao et al. (2017) distributed under the Creative Commons Attribution Licens].

Epidermal growth factor (EGF) and human adipose cell-derived stem cell exosome-loaded microcapsules integrated with collagen hydrogel can effectively show tissue regeneration and also restoration of blood perfusion in diabetic wounds (Cao et al., 2017). In the previously published literature, it has been found that adipose-derived MSC exosomes incorporated in freeze–thaw-based polypeptide-based hydrogel possess self-healing, antibacterial, and exosome release characteristics (Shen et al., 2016). These properties are useful in promoting wound healing by enhancing cell proliferation, neovascularization, re-epithelialization, and collagen remodeling at the wound site (Wang et al., 2019). In another recent tailoring approach, the cells are genetically engineered with transfection and co-culture to synthesize exosomes containing long non-coding RNA H19 that helps promote wound healing in DFU mediated by upregulation of PTEN through miRNA-152-3p (Li et al., 2020). Figure 3 demonstrates the paracrine effect of BMSCs in treatment of DFUs mediated *via* EVs. These tailoring approaches of exosomes may help provide promising results in the healing of DFUs associated with bacteria. The current work encourages the implication of differential centrifugation and ultracentrifugation method for isolation of EVs from spent media or any other sources. The reason for recommending these two methods is due to their low cost and easy installation in any lab/clinic. Moreover, the genetic engineering approach endogenous modification is suitable for modification of EVs if they are used for delivering genes of interest. The modified EVs can be easily used in the treatment of ulcers/wounds associated with the DM. For instance, DFUs associated with bacteria need antibacterial and regenerative therapy. EVs, if modified for gene delivery (for initiating regeneration of damaged skin) and drug (antibiotics/antibacterial), can fulfill the purpose of therapeutic intervention.

## Pathogenesis of Bacteria-Associated DFU

Diabetes mellitus is characterized by high blood glucose level and neuropathy that slow down the wound healing process. These slow-healing wounds are vulnerable to bacterial infections (Buch et al., 2019). These diabetic wounds and foot ulcers become chronic due to microbe habitat on the wound site (Bjarnsholt et al., 2008). This continuous growth of bacteria (both aerobes and anaerobes) on the wound site produces biofilm, which exhibits resistance toward antibiotics that in turn causes a problem in the treatment of these wounds (Shiau and Wu, 1998; Bridier et al., 2011). It has been observed that *Staphylococcus aureus* is among the most common bacteria that are prevalent in DFUs (Kalan et al., 2019). Moreover, other bacteria causing DFUs includes β-hemolytic streptococci, *S. aureus*, *S. saprophyticus*, *S. epidermis*, *Streptococcus pyogenes*, *S. mutans*, *P. aeruginosa*, *Bacillus subtilis*, *Proteus* species, *Escherichia coli*, and *Klebsiella pneumoniae*. The anaerobic bacteria include *Peptostreptococcus* species, anaerobic streptococci, *Bacteroides fragilis*, and *Clostridium* species (Lipsky et al., 2012; Richard et al., 2012; Kalan et al., 2019). Bacterial biofilms of diabetic wounds and DFUs are protected from various stresses, including antibiotics and immune responses. Biofilm production involves the uncontrolled growth of sessile and planktonic bacteria that grow continuously on themselves to form a layer that is termed biofilm. Treatment of biofilms is also a major health concern as emphasized by the World Health Organization (WHO), as it contributes to the development of antimicrobial resistance toward antibiotics. Clinicians and researchers are focusing on the promising alternative treatment approaches to the use of antibiotics in reducing bacterial infections. Natural sources such as plant-derived extracts, polyphenols, anti-sense RNA, and stem cell-derived exosomes might be the prospective alternative therapies to manage DFUs and diabetic wounds. Several emerging technologies identify the risk assessment associated with DFUs, including laser Doppler flowmetry, infrared thermography, ultrasound indentation tests (elastography), and plantar pressure and pressure gradient system (Lung et al., 2020). These technologies may be helpful in the screening of risk in DFUs, so that treatment approaches may be customized accordingly.

## Pathophysiology of Controlling Bacteria-Associated DFU Using MSC-Derived Exosomes

Extracellular vesicles are the key component of cell-to-cell communication that facilitates transfer of internalized cargo, including proteins, nucleic acids, and other biological factors. EVs are known to play an active role in pathological conditions like kidney injury, inflammatory disorders, wound healing, and regeneration, along with several therapeutic and diagnostic characteristics. A previously published study demonstrated that EVs possess antimicrobial peptides (AMPs) (Hiemstra et al., 2014). EVs were also reported to contain lysozyme C, dermcidin, mucin-1, calprotectin, and myeloperoxidase and to have a bactericidal effect. In one of the recent studies conducted *in vitro* on urinary exosomes, it was found that these exosomes showed a bactericidal effect against *E. coli* (Francisca et al., 2017). The same research concluded that nasal lavage fluid-derived exosomes showed defense against pathogens and allergens (Francisca et al., 2017).

In another study, it was found that EVs released from biliary and intestinal epithelium luminal contain AMPs along with LL-37 and hBD-2 that activate the toll-like receptor (TLR)-4 signaling cascade and contribute toward antimicrobial defense (Hu et al., 2013). In the past few years, MSC-derived EVs have been explored for therapeutic, diagnostic, and anti-inflammatory roles in several pre-clinical trials. In one of the published reports, it was found that MVs secreted by BMSCs are efficient in the treatment of acute lung injury (ALI) caused by *E. coli* endotoxins *via* transfer of keratinocyte growth factor (KGF) mRNA from the MVs to damaged lung endothelium and alveolar epithelium (Zhu et al., 2014). In another animal study conducted on a bacterial pneumonia mouse model, it was demonstrated that BMSC-extracted MVs showed significant survival and lessen the influx of inflammatory cells (Monsel et al., 2015). In another study, the antimicrobial effect of MSC-derived EVs was demonstrated, which is mediated by the transfer of mitochondria into the target cells that in turn increases the phagocytosis of macrophages (Islam et al., 2001). Several *in vivo* clinical trials demonstrated the antibacterial effect of MSC-derived EVs (Krasnodembskaya et al., 2010; Harman et al., 2017; Cortés-Araya et al., 2018). However, more studies and clinical trials are needed to establish the significant role of MSC-derived EVs as antimicrobial agent. This antimicrobial effect of EVs can be explored and serve as a prospective therapy for the treatment of diabetic wounds and DFUs.

## Safety and Toxicology Considerations of Exosomes

Extracellular vesicles are known to be the safest therapeutic approach for both pre-clinical and clinical use. There were no signs of toxicity observed in previously published literature except that some human cell-derived EVs possess the potential to elicit an immune response, which is a positive sign for using EVs as cell-free therapeutic approach in DFUs (Zhu et al., 2017). In one study, C57BL/6 mice were given EVs for 3 weeks *via* intravenous and intraperitoneal administration, and no toxicity was observed with slight changes in expression of immune markers (Zhu et al., 2017). In another murine study, BMSC-derived engineered exosome (iExosomes) administration did not produce any toxicity and adverse immune reactions (Mendt et al., 2018). The engineered approaches for EVs mentioned in the present work suggest that EVs are a safe and non-toxic method for delivering cargo compared to cationic lipids, viral vectors, and polymer-based methods (Mendt et al., 2018). Moreover, long-term pre-clinical and clinical studies are needed to further evaluate the toxicological and immunological profile of engineered EVs (Table 2).

**TABLE 2:** Different aspects of exosomes.

Feature	Exosome	Apoptotic body	MV
*Size*	Homologous 30–100 nm	Heterogeneous 1–5 μm	Heterogeneous 100–1000 nm
*Markers*	Membrane impermeable (PI negative) CD63, TSG101, Alix, flottilin	Membrane permeable (PI positive) Annexin V, DNA, histones	Membrane impermeable (PI negative) integrin, selectin, flotillin-2
*Density*	1.13–1.19 g/mL	1.16–1.28 g/mL	1.25–1.30 g/mL
*Contents*	Protein, lipid, different RNA species, and DNA	Cytosolic content (protein, RNAs, fragmented DNA) and cellular organelles	Protein, lipid, different RNA species, and DNA
*Determinant of controlled contents*	The cellular origin and physiological state of the cell	The cellular origin and stimuli	No direct correlation
*Lipids*	A major sorting of lipidic molecules from the parental cells (include BMP)	Characterized by phosphatidylserine externalization	The lipid contents are primarily derived from plasma membrane, and resemble the parental cells (without BMP)
*Origin*	Multivesicular bodies fusion with plasmatic membrane	Cellular debris, plasma membrane blebbing during cell apoptosis	Direct outward budding or blebbing from the plasma membrane
*Mechanism of release*	Constitutive or inducible, depending on the cell type of origin	Rho-associated kinase I and myosin ATPase activity	Relocation of phospholipids to the outer membrane, cytoskeleton rearrangements, generation of membrane curvature, and vesicle release
*Detection methods*	Electron microscopy, Western blot for exosome enriched markers	Flow cytometry, electron microscopy,	Flow cytometry, electron microscopy
*Isolation methods*	Ultracentrifugation (100,000–200,000 × *g*) filtration, density gradient Immunoprecipitation, Immune affinity capture and ExoQuick precipitation methods	Ultracentrifugation (10,000–20,000 × *g*)	No standardized methods
*Modification methods*	Incubation, Sonication, Extrusion, Freeze thaw, Electroporation, Chemical transfection, Genetic engineering		
*Size determination and quantification*	Dynamic light scattering Nanoparticle tracking analysis Surface plasmon resonance		

*MV, microvesicle; BMP, bone morphogenetic protein; PI, propidium iodide. Adopted from Zhang et al. (2019) distributed under the Creative Commons Attribution 4.0 International License.*

## Conclusion

Extracellular vesicles are emerging as new therapeutics in the management of diseases, regeneration of tissue, and diagnostic markers. The heterogeneity and complexity with the ability of modification under a physiological and pathological environment make them interesting candidates for implication in the biological field. Exosomes have the potential to treat various diseases due to flexibility of loading diverse drugs and modifications. Exosomes can be used for detection, diagnosis, and treatment only because of their tendency of modification in the membrane. Moreover, MSC-derived exosomes are primarily exploited for regenerative medicine. Despite the fact that many advances in the modification approach of exosomes are currently being practiced; one of the most significant challenge with these vesicles is their inefficient production at a large scale for clinical use following GMP/GCP guidelines. MSC-derived exosomes are a rich source of AMPs along with other anti-bactericidal factors, which opens up the window of treating DFUs caused by microorganisms including *S. aureus*, *S. saprophyticus*, *S. epidermis*, *S. pyogenes*, *S. mutans*, *P. aeruginosa*, *B. subtilis*, *Proteus* species, *E. coli*, and *K. pneumoniae*. The potential bactericidal efficacy of the MSC-derived exosomes can be amplified through modification of cell conditioning medium and drug loading approach. AMP-encapsulated exosomes can be exploited further for clinical trials to treat DFUs associated with microbes. Notable EV-based management therapies promote wound/ulcer healing along with minimal scarring without ethical issues and conflicts. Future studies including pre-clinical and clinical trials are required to explore the therapeutic and anti-microbial effect of the MSC-derived exosomes. These EVs can be exploited in designing wound dressings that might be prospectively used in the treatment of DFUs associated with bacteria.

## Author’s Note

AR, PT, and KG are currently involved in COVID-19 testing duties.

## Author Contributions

AR, BM, SB, KG, QM, and AS are involved in manuscript writing, conceptualization, and data analysis. PT, G-BJ, PS, MS, and JA supervised and reviewed the manuscript. All the authors have read and agreed to the published version of the manuscript.

## Conflict of Interest

The authors declare that the research was conducted in the absence of any commercial or financial relationships that could be construed as a potential conflict of interest.

## Publisher’s Note

All claims expressed in this article are solely those of the authors and do not necessarily represent those of their affiliated organizations, or those of the publisher, the editors and the reviewers. Any product that may be evaluated in this article, or claim that may be made by its manufacturer, is not guaranteed or endorsed by the publisher.
